# Supplementary material for: Strength and Microstructure Assessment of Partially Replaced Ordinary Portland Cement and Calcium Sulfoaluminate Cement with Pozzolans and Spent Coffee Grounds
Source: Materials (Basel). 2023 Jul 14;16(14):5006. doi: 10.3390/ma16145006 (PMC10384487; doi:10.3390/ma16145006)
Supplement: Supplementary file 1 [file materials-16-05006-s001.zip › materials-2471067-supplementary.pdf]

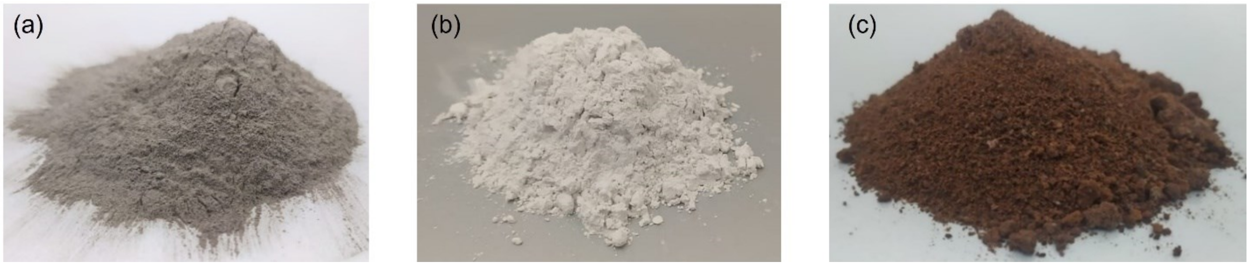

Figure S1. Images of pozzolans used in the investigation (**a**) fly ash (**b**) volcanic ash (**c**) dried spent coffee grounds.

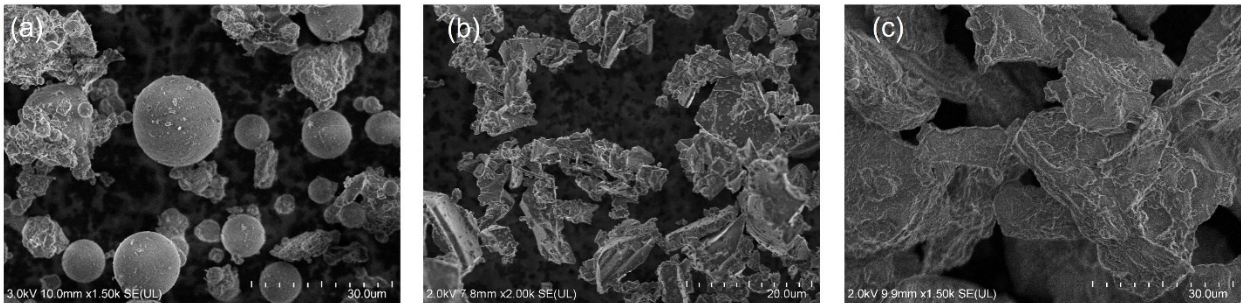

Figure S2. SEM images of pozzolans used in the investigation (**a**) fly ash (**b**) volcanic ash (**c**) dried spent coffee grounds.
